# 38351 Immune-related adverse events in cancer patients receiving immune checkpoint inhibitors

**DOI:** 10.1017/cts.2021.698

**Published:** 2021-03-30

**Authors:** Margaret Byrnethew Lucas, Lori Pai, Janis Breeze, Susan Parsons

**Affiliations:** Tufts Medical Center

## Abstract

ABSTRACT IMPACT: The existing literature describing immune-related adverse events (irAE) has predominantly focused on clinical trial populations, which may not be representative of the broader population receiving immune checkpoint inhibitors (ICI), so we sought to perform a comprehensive evaluation of irAE in a real-world population of cancer patients being treated with ICI. OBJECTIVES/GOALS: With a cohort of patients with malignancy treated with ICI, we characterized incidence, severity, timecourse of ir-AE. We sought to inform providers who prescribe ICI to recognize the clinical burden of irAE in an effort to more effectively communicate the benefits and risks of ICI with patients. METHODS/STUDY POPULATION: After obtaining approval from the institutional review board, we used a pharmacy database to identify adult cancer patients treated with an ICI between January 2014 and October 2018. We used electronic medical records to obtain baseline variables. Each patient was followed at each clinic visit for 12 months for development of a physician-reported irAE.

For irAE, site and grade were recorded as documented by the provider. At diagnosis and each follow-up visit, we collected: 1) adjustments to ICI, immunosuppression, and hormone therapy.

Continuous variables were summarized using mean and standard deviation (SD) or median and interquartile range (IQR). Categorical variables were summarized using frequencies and percentages. Time to development and resolution of irAE were calculated using Kaplan-Meier curves. RESULTS/ANTICIPATED RESULTS: Among 131 patients, two-thirds were men, and 60% were Caucasian with a mean age was 65 years. Nearly 40% had an Eastern Cooperative Oncology Group performance score of 2 or higher. A small proportion (3.1%) had an autoimmune disorder. Nearly half had lung cancer (49.6%), and several had received radiation (33.6%). Over 70% were former or current smokers. In total, 57 patients (43.5%) developed an irAE, resultng in a total of 95 irAE, at a median of 208 days (126 days, not reached). The most common irAE included dermatitis, thyroiditis, pneumonitis, and hepatitis. Of 95 irAE, half were grade 1, 30% were grade 2, and nearly 20% were grade 3 or higher. Median time to resolution was 85 days (56-183 days). DISCUSSION/SIGNIFICANCE OF FINDINGS: This study demonstrates that irAE are clinically impactful in this relatively unfit, medically complex population, which may be a more accurate reflection of patients receiving ICI in everyday practice. This study showcases that this population is susceptible to irAE, although this needs to be examined in larger, prospective trials.

